# Alteration of Skin Wound Healing in Keratinocyte-Specific Mediator Complex Subunit 1 Null Mice

**DOI:** 10.1371/journal.pone.0102271

**Published:** 2014-08-14

**Authors:** Fumihito Noguchi, Takeshi Nakajima, Shigeki Inui, Janardan K. Reddy, Satoshi Itami

**Affiliations:** 1 Department of Regenerative Dermatology, Graduate School of Medicine, Osaka University, Osaka, Japan; 2 Department of Dermatology, Osaka General Medical Center, Osaka, Japan; 3 Department of Pathology, Feinberg School of Medicine, Northwestern University, Chicago, Illinois, United States of America; University of Maryland School of Medicine, United States of America

## Abstract

MED1 (Mediator complex subunit 1) is a co-activator of various transcription factors that function in multiple transcriptional pathways. We have already established keratinocyte-specific MED1 null mice (*Med1^epi−/−^*) that develop epidermal hyperplasia. Herein, to investigate the function(s) of MED1 in skin wound healing, full-thickness skin wounds were generated in *Med1^epi−/−^* and age-matched wild-type mice and the healing process was analyzed. Macroscopic wound closure and the re-epithelialization rate were accelerated in 8-week-old *Med1^epi−/−^* mice compared with age-matched wild-type mice. Increased lengths of migrating epithelial tongues and numbers of Ki67-positive cells at the wounded epidermis were observed in 8-week-old *Med1^epi−/−^* mice, whereas wound contraction and the area of α-SMA-positive myofibroblasts in the granulation tissue were unaffected. Migration was enhanced in *Med1^epi−/−^* keratinocytes compared with wild-type keratinocytes *in vitro*. Immunoblotting revealed that the expression of follistatin was significantly decreased in *Med1^epi−/−^* keratinocytes. Moreover, the mitogen-activated protein kinase pathway was enhanced before and after treatment of *Med1^epi−/−^* keratinocytes with activin A *in vitro*. Cell-cycle analysis showed an increased ratio of S phase cells after activin A treatment of *Med1^epi−/−^* keratinocytes compared with wild-type keratinocytes. These findings indicate that the activin-follistatin system is involved in this acceleration of skin wound healing in 8-week-old *Med1^epi−/−^* mice. On the other hand, skin wound healing in 6-month-old *Med1^epi−/−^* mice was significantly delayed with decreased numbers of Ki67-positive cells at the wounded epidermis as well as BrdU-positive label retaining cells in hair follicles compared with age-matched wild-type mice. These results agree with our previous observation that hair follicle bulge stem cells are reduced in older *Med1^epi−/−^* mice, indicating a decreased contribution of hair follicle stem cells to epidermal regeneration after wounding in 6-month-old *Med1^epi−/−^* mice. This study sheds light on the novel function of MED1 in keratinocytes and suggests a possible new therapeutic approach for skin wound healing and aging.

## Introduction

The wound healing process is divided into three phases: an inflammatory phase, a proliferative phase and a remodeling phase [1], [2]. The inflammatory phase occurs immediately after injury. Tissue damage initially causes the disruption of vascular vessels and extravasation, followed by the production of a temporary platelet plug and a fibrin clot which stops bleeding and supplies a transient anchorage for subsequently infiltrating inflammatory cells. Next, during the proliferative phase, which occurs several days after tissue damage, keratinocytes and endothelial cells proliferate and migrate to the wound, resulting in re-epithelialization and angiogenesis. Finally, in the remodeling phase, some fibroblasts are stimulated by macrophages to differentiate into myofibroblasts, causing wound contraction. During this phase, production of the extracellular matrix, including collagen, proteoglycan and fibronectin, is increased, which results in the formation of a mature scar [3], [4]. All of these events require the orchestrated efforts of different types of cells. Failure in any of these phases of the wound healing process can lead to chronic wounds, hypertrophic scars and/or wound-related tumor formation [5].

Mediator complex subunit 1 (MED1) is integrated into the Mediator complex as a coactivator of various transcription factors, including nuclear receptors, p53 and BRCA1 [6], [7]. MED1 has also been reported to play critical roles in regulating hair cycling and epidermal proliferation [8]. Previously, we established keratinocyte-specific MED1-null (*Med1^epi−/−^*) mice and characterized the roles of MED1 in regulating the proliferation of keratinocytes and the maintenance of hair follicle bulge stem cells [9]. In this study, we investigated the process of wound healing in *Med1^epi−/−^* skin and analyzed the underlying mechanisms, including the activin-follistatin system and epithelial stem cells.

## Results

### Skin wound healing is accelerated in 8-week-old *Med1^epi−/−^* mice

**Figure 1:**
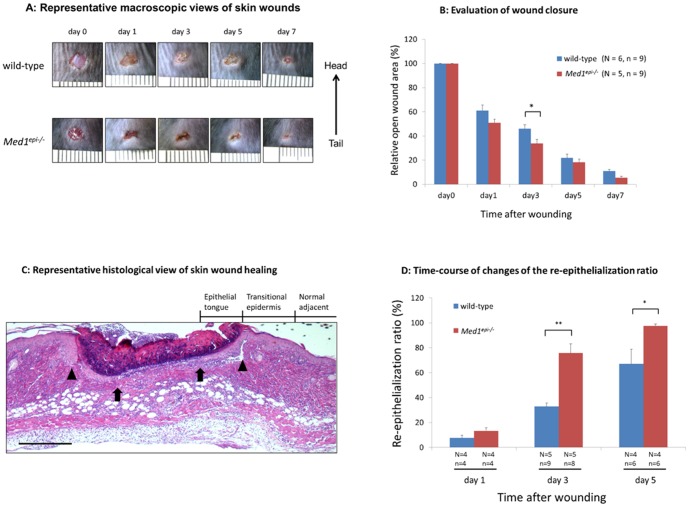
Skin wound healing is accelerated in 8-week-old *Med1^epi−/−^* mice. A: Representative macroscopic views of skin wounds on days 1, 3, 5 and 7 after wounding in 8 week old wild-type and *Med1^epi−/−^* mice. Full-thickness wounds (4 mm in diameter) were made on the middle of the backs of mice to synchronize tension and wound healing was monitored by taking digital photographs. Note the acceleration of wound healing in *Med1^epi−/−^* mice. B: Evaluation of wound closure by morphometrical analysis of the wound areas. The % of the wound area to the initial area was calculated from the photographs. N = number of mice; n = number of measurements. Bars = means ± SE. *P<0.05. C: Representative histological view of skin wound healing on day 3. Arrowheads and arrows indicate original wound edges and re-epithelialized leading edges, respectively. Scale bar = 500 µm. D: Time-course of changes of the re-epithelialization ratio after wounding in wild-type and *Med1^epi−/−^* mice. The % re-epithelialization was calculated by measuring the distance between the leading edges and the width between original wound edges as described in the Materials and Methods. N = number of mice; n = number of sections. Bars = means ± SE. *P<0.05, **P<0.01.

To study the effect of MED1 depletion in keratinocytes on the skin wound healing process, we created full-thickness circular excisional wounds on the backs of 8-week-old *Med1^epi−/−^* mice and wild-type (*Med1^+/+^*) mice and observed the healing process through days 1 to 7 after injury (Figure 1A). Macroscopic evaluation revealed that wound closure of *Med1^epi−/−^* mice was significantly accelerated on day 3 after injury compared with wild-type mice (Figure 1B, p<0.05). Next, we performed skin biopsies at these wound sites on days 1, 3 and 5 after injury and evaluated the skin wound healing process microscopically (Figure 1C). Hematoxylin and eosin (H&E) staining of wound sites indicated that re-epithelialization after wounding was significantly enhanced in *Med1^epi−/−^* mice on days 3 (p<0.01) and 5 (p<0.05) compared with wild-type mice (Figure 1D).

### Migrating epithelial tongues are elongated and the proliferation of keratinocytes is accelerated in 8-week-old *Med1^epi−/−^* mice

**Figure 2:**
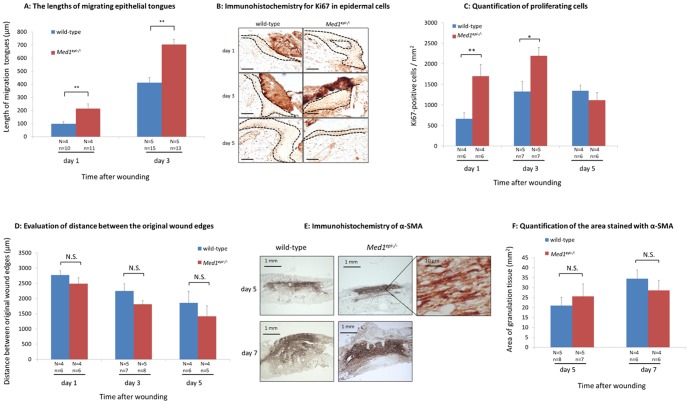
Migrating epithelial tongues are elongated and the proliferation of keratinocytes is enhanced in 8-week-old *Med1^epi−/−^* mice. A: The lengths of migrating epithelial tongues were measured on days 1 and 3 after injury in 8-week-old wild-type and *Med1^epi−/−^* mice. N = number of mice; n = number of measurements. Bars = means ± SE. **P<0.01. B: Analysis of keratinocyte proliferation at the re-epithelialized leading edges in 8-week-old wild-type and *Med1^epi−/−^* mice. Images show representative high-power fields of immunohistochemistry for Ki67 in epidermal cells in the transitional epidermis and in the migrating epithelial tongues on days 1, 3 and 5 after injury. Scale bar = 50 µm. C: Quantification of proliferating cells on days 1, 3 and 5 after injury. Ki67-positive cells were counted in the transitional epidermis and the epithelial tongues [2] of wound sites in 8-week-old wild-type and *Med1^epi−/−^* mice and were related to the area of the same part of the epidermis. N = number of mice; n = number of measurements. Bars = means ± SE. *P<0.05, **P<0.01. D: Evaluation of distance between the original wound edges in 8-week-old wild-type and *Med1^epi−/−^* mice on days 1, 3 and 5 after injury. N = number of mice; n = number of sections. Bars = means ± SE. N.S., not significant. E: Immunohistochemistry of α-SMA for the detection of myofibroblasts in the granulation tissue on days 5 and 7 after wounding. F: The area stained with α-SMA was determined by planimetric image analysis using ImageJ software. N = number of mice; n = number of sections. Bars = means ± SE. N.S., not significant.

To investigate the mechanism(s) underlying the accelerated wound healing in 8-week-old *Med1^epi−/−^* mice, we next compared the lengths of migrating epithelial tongues and observed a significant elongation in *Med1^epi−/−^* mice on days 1 (p<0.01) and 3 (p<0.01) after injury (Figure 2A). Moreover, Ki67 immunostaining in the aforementioned period clearly showed that the number of Ki67-positive keratinocytes was increased at the transitional epidermis and the epithelial tongues were longer in 8-week-old *Med1^epi−/−^* mice on days 1 (p<0.01) and 3 (p<0.05) after injury compared with those in age-matched wild-type mice (Figure 2B and C), indicating the acceleration of keratinocyte proliferation by Med1 knockout. We have previously reported that Ki67-positive proliferating keratinocytes in unwounded skin of 8-week-old *Med1^epi−/−^* mice were 1.57 times more frequently observed than in wild-type mice [9]. The number of Ki67-positive proliferating keratinocytes in the wounded epidermis was increased by 2.56-fold in 8-week-old *Med1^epi−/−^* mice compared with wild-type mice on day 1 after injury (Figure 2C). These findings suggest that enhanced keratinocyte migration and proliferation contribute to the acceleration of skin wound healing in 8-week-old *Med1^epi−/−^* mice.

As wound contraction also significantly contributes to the wound healing process, the distance between the original wound edges was microscopically measured to precisely evaluate the contraction of wounds in *Med1^epi−/−^* skin. The original wound edges were determined as the start sites of re-epithelialization. As shown in Figure 2D, there was no significant difference in wound contraction between *Med1^epi−/−^* and wild-type mice. Further, because myofibroblasts play pivotal roles in granulation and scar formation as well as in wound contraction, we investigated dermal myofibroblasts in the wound sites on days 5 and 7 after wounding (Figure 2E). Myofibroblasts were identified by staining for α-SMA. α-SMA-positive myofibroblasts were similarly distributed in 8-week-old *Med1^epi−/−^* and wild-type mice (Figure 2F), suggesting that granulation and scar formation were not affected in 8-week-old *Med1^epi−/−^* mice.

### Follistatin expression is decreased and the MAPK pathway is activated in *Med1^epi−/−^* keratinocytes *in vitro*

**Figure 3:**
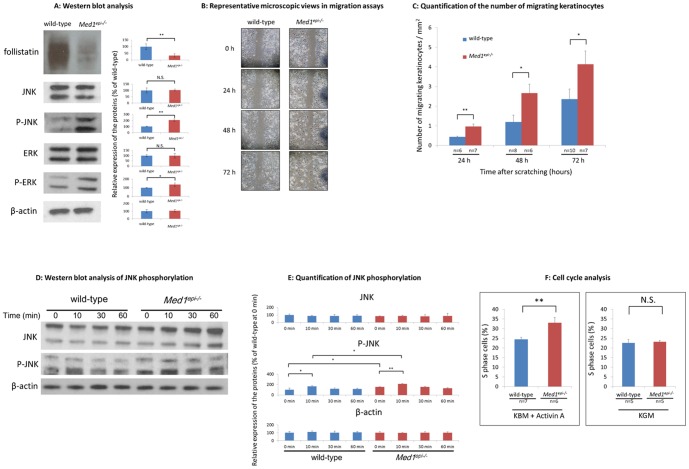
Follistatin expression is decreased and the MAPK signaling pathway is activated in *Med1^epi−/−^* mice keratinocytes *in vitro*. A: Western blot analysis of follistatin, JNK, phospho-JNK, ERK and phospho-ERK in wild-type and *Med1^epi−/−^* keratinocytes (left). Quantification of the expression of each protein (right) (n = 3). Bars = means ± SE. *P<0.05, **P<0.01. B: Representative microscopic views in migration assays. Keratinocytes were cultured in KBM to form confluent monolayers and then were serum deprived for 24 h. The cells were subsequently incubated with mitomycin C (0.5 mg/ml) for 2 h and were then scratched with a p200 pipette tip, followed by incubation in KBM for 72 h. The cells were analyzed by phase contrast microscopy and were photographed at the indicated time points. C: Quantification of the number of migrating keratinocytes. The number of cells which had migrated into the wounded space at the indicated time points was counted microscopically and related to the wounded area. Bars = means ± SE. *P<0.05, **P<0.01. D: Western blot analysis of JNK phosphorylation after treatment with activin A (5 ng/mL). The level of JNK phosphorylation reached the highest value at 10 min after activin A treatment in wild-type and in *Med1^epi−/−^* keratinocytes cultured in KBM. Note that both the peak and the basal level of JNK phosphorylation were enhanced in *MED1^epi−/−^* keratinocytes compared with wild-type keratinocytes. E: Quantification of JNK phosphorylation after administration of activin A in *Med1^epi−/−^* and wild-type keratinocytes (n = 3). Bars = means ± SE. *P<0.05, **P<0.01. F: Cell cycle analysis of *Med1^epi−/−^* and wild-type keratinocytes. Cultured in KBM with acitivin A (5 ng/mL), *Med1^epi−/−^* keratinocytes showed an increased ratio of S phase cells compared with wild-type keratinocytes (left) while no difference was observed when those cells were cultured in KGM (right). Bars = means ± SE. **P<0.01, N.S., not significant.

Our previous microarray study comparing gene expression profiles between *Med1^epi−/−^* and wild-type keratinocytes [9], which is deposited in the GEO repository (http://www.ncbi.nlm.nih.gov/geo) under the accession number GSE35406, revealed that the expression of follistatin is significantly suppressed in *Med1^epi−/−^* keratinocytes, while the expression of activin, a target of follistatin, as well as activin receptors is not altered. Consistent with this previous data, the expression of follistatin in *Med1^epi−/−^* keratinocytes was significantly decreased compared with wild-type keratinocytes (Figure 3A). In the activin-follistatin system crucial for wound repair [10], [11], follistatin sequesters and inhibits activin. On the other hand, activin secreted from keratinocytes and fibroblasts during the wound healing process [12], [13] activates the MAPK pathway in keratinocytes, influencing their proliferation as well as their migration [13]–[17]. These facts prompted us to study whether the MAPK pathway is activated in *Med1^epi−/−^* keratinocytes. The phosphorylation of JNK as well as ERK was enhanced in *Med1^epi−/−^* -derived keratinocytes (Figure 3A) compared with wild-type keratinocytes but the phosphorylation of p38 was not apparently enhanced in *Med1^epi−/−^* keratinocytes (data not shown). These results indicated that endogenous activin secreted from keratinocytes *in vitro* can robustly activate the MAPK pathway in an autocrine manner in *Med1^epi−/−^* keratinocytes, where follistatin expression was decreased.

### Migration is enhanced in *Med1^epi−/−^* keratinocytes *in vitro*

Next, to test the effect of endogenous activin secreted from keratinocytes on the migration of *Med1^epi−/−^* keratinocytes, we performed an *in vitro* wound healing assay in medium without growth factors (Figure 3B). Under these conditions, *Med1^epi−/−^* keratinocytes showed enhanced motility at 24 h, 48 h and 72 h after wounding compared with wild-type keratinocytes (Figure 3C), indicating that endogenous activin secreted from keratinocytes *in vitro* may activate migration more intensely in *Med1^epi−/−^* keratinocytes, conceivably because their expression of follistatin was decreased.

### JNK phosphorylation in *Med1^epi−/−^* keratinocytes is augmented by exogenous activin A *in vitro*

It has been reported that activin A is mainly secreted from dermal fibroblasts and acts on keratinocytes in a paracrine manner, contributing to skin homeostasis, wound healing and hair cycling [13]. Accordingly, several reports have suggested that exogenous activin can enhance the proliferation and migration of keratinocytes by activating the MAPK pathway [15]–[17]. Therefore, we next examined the activation of JNK in *Med1^epi−/−^* keratinocytes by exogenous activin A. As shown in Figure 3D, activin A caused an immediate and transient JNK phosphorylation, which was detectable at 10 min after the treatment and was reduced to the basal level at 30 min in *Med1^epi−/−^* and in wild-type keratinocytes. The peak level as well as the basal level of phosphorylation of JNK in *Med1^epi−/−^* keratinocytes was augmented compared with wild-type keratinocytes (Figure 3E), suggesting that exogenous as well as endogenous activin A enhances JNK phosphorylation in *Med1^epi−/−^* keratinocytes because of their decreased expression of follistatin.

### Exogenous activin A increases the percentage of *Med1^epi−/−^* keratinocytes in S-phase

We next asked if exogenous activin A also influences the cell cycle of *Med1^epi−/−^* keratinocytes. To optimize the readout, the cells were cultured in KBM and starved for 24 h, were subsequently treated with activin A for 24 h and then were finally subjected to cell cycle analysis. The results showed that the S-phase percentage of activin A-treated *Med1^epi−/−^* keratinocytes cultured in KBM was significantly higher than activin A-treated wild-type keratinocytes (Figure 3F, left, p<0.01). On the other hand, cell cycle phases were similar in activin A-treated *Med1^epi−/−^* and wild-type keratinocytes when cultured in keratinocyte growth medium (KGM) containing numerous growth promoters (Figure 3F, right). This is probably because the excess growth promoters in the KGM masked the endogenous activin A effect.

Together these data suggest that the wound healing acceleration in 8-week-old *Med1^epi−/−^* mice could be ascribed to the alteration of follistatin-activin balance in the wound sites, which activates MAPK signaling and keratinocyte proliferation and migration.

### Skin wound healing in old *Med1^epi−/−^* mice is delayed

**Figure 4:**
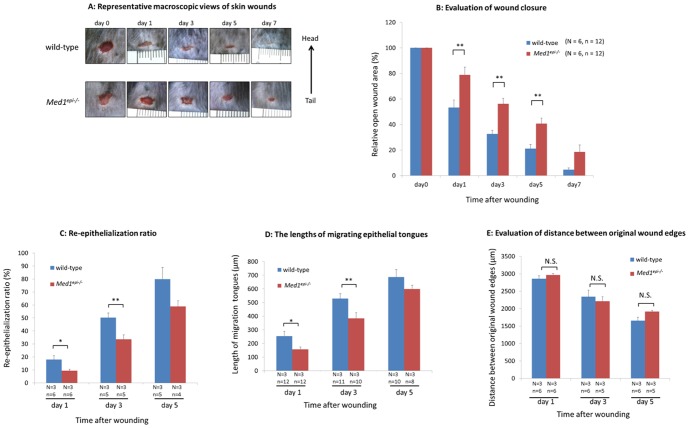
Skin wound healing is delayed in 6 month old *Med1^epi−/−^* mice. A: Representative macroscopic views of skin wounds on days 1, 3, 5 and 7 after wounding in 6-month-old wild-type and *Med1^epi−/−^* mice. Full-thickness wounds (4 mm in diameter) were made on the middle of the back skins of mice and wound healing was monitored by taking digital photographs. Note that wound healing in 6-month-old *Med1^epi−/−^* mice was significantly delayed compared with the age-matched wild-type mice. B: Evaluation of wound closure by morphometrical analysis of the wound areas in 6-month-old wild-type and *Med1^epi−/−^* mice. The % of the wound area to the initial area was calculated from the photographs. N = number of mice; n = number of measurements. Bars = means ± SE. **P<0.01. C: Re-epithelialization ratio on days 1, 3 and 5 after wounding in 6-month-old wild-type and *Med1^epi−/−^* mice. The % re-epithelialization was calculated as mentioned above. N = number of mice; n = number of sections. Bars = means ± SE. *P<0.05, **P<0.01. D: The lengths of migrating epithelial tongues were measured on days 1, 3 and 5 after injury in 6-month-old wild-type and *Med1^epi−/−^* mice. N = number of mice; n = number of measurements. Bars = means ± SE. *P<0.05, **P<0.01. E: Evaluation of distance between original wound edges in 6-month-old wild-type and *Med1^epi−/−^* mice on days 1, 3 and 5 after injury. N = number of mice; n = number of sections. Bars = means ± SE. N.S., not significant.

**Figure 5:**
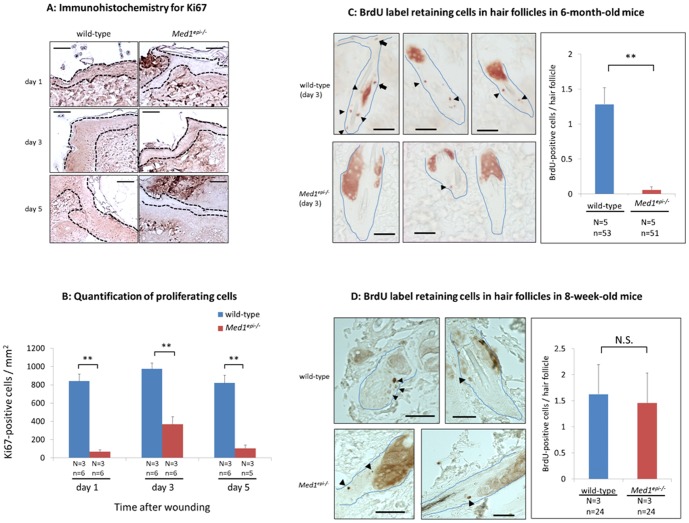
Proliferative keratinocytes and BrdU-positive label retaining cells are decreased in *Med1^epi−/−^* mice. A: Images show representative high-power fields of immunohistochemistry for Ki67 in 6-month-old wild-type and *Med1^epi−/−^* mice keratinocytes in the transitional epidermis and the migrating epithelial tongues on days 1, 3 and 5 after injury. Scale bars = 50 µm. B: Quantification of proliferating cells on days 1, 3 and 5 after injury. Ki67-positive cells were counted in the transitional epidermis and the epithelial tongues of wound sites in 6-month-old wild-type and *Med1^epi−/−^* mice and were related to the area of the same part of the epidermis. N = number of mice; n = number of measurements. Bars = means ± SE. **P<0.01. C: BrdU-positive slow-cycling label retaining cells in hair follicles in 6-month-old wild-type and *Med1^epi−/−^* mice were detected on day 3 after injury (left). Arrowheads, BrdU-positive label retaining cells; Arrows, BrdU-positive label retaining cells migrating into epidermis adjacent to the wounds. The number of BrdU-positive cells in hair follicles was significantly decreased in 6-month-old *Med1^epi−/−^* mice compared with age-matched wild-type mice (right). Scale bars = 25 µm. N = number of mice; n = number of hair follicles. Bars = means ± SE. **P<0.01. D: BrdU label retaining cells in hair follicles in 8-week-old wild-type and *Med1^epi−/−^* mice (left). Arrowheads, BrdU-positive label retaining cells. The number of BrdU-positive cells in hair follicles in 8-week-old *Med1^epi−/−^* mice was comparable with age-matched wild-type mice (right). Scale bars = 25 µm. N = number of mice; n = number of hair follicles. Bars = means ± SE. N.S., not significant.

Next, to assess the skin wound healing process in older *Med1^epi−/−^* mice, we performed wound healing assays in 6-month-old wild-type and *Med1^epi−/−^* mice. As demonstrated in Figure 4A and 4B, the wound healing process was significantly delayed on days 1 (p<0.01), 3 (p<0.05) and 5 (p<0.01) after injury in 6-month-old *Med1^epi−/−^* mice, compared with wild-type mice. In line with this observation, the re-epithelialization ratio was significantly decreased on days 1 (p<0.05) and 3 (p<0.01) after injury in 6-month-old *Med1^epi−/−^* mice (Figure 4C). The lengths of migrating epithelial tongues were correspondingly decreased on days 1 (p<0.05) and 3 (p<0.05) after injury in 6-month-old *Med1^epi−/−^* mice (Figure 4D), while no significant difference was observed in the wound contraction between 6-month-old wild-type and *Med1^epi−/−^* mice (Figure 4E). Compared with age-matched wild-type mice, Ki67-positive proliferating keratinocytes were decreased in 6-month-old *Med1^epi−/−^* mice per area of transitional epidermis and the epithelial tongue in wound sites on days 1 (p<0.01), 3 (p<0.01) and 5 (p<0.01) after the injury (Figure 5A and B). Immunohistochemical staining for follistatin revealed no difference in the expression of follistatin between 8-week-old and 6-month-old *Med1^epi−/−^* mice (data not shown), suggesting that a mechanism(s) other than the activin-follistatin system could influence this impediment of wound healing in 6-month-old *Med1^epi−/−^* mice.

### There is less contribution of BrdU-positive label retaining cells in hair follicles to cutaneous wound healing in old *Med1^epi−/−^* mice

It has been reported that, after epidermal injury, hair follicle stem cells give rise to short-lived transient amplifying cells, which migrate into the wound epithelium and promote the epidermal regeneration [18]–[27]. In our previous study, CD34-positive and keratin 15-positive hair follicle bulge stem cells decreased in *Med1^epi−/−^* mice after several months of age, resulting in sparse hair in older *Med1^epi−/−^* mice [9]. Therefore, we hypothesized that the delay of skin wound healing in the older *Med1^epi−/−^* mice can be attributed to the possible reduction of hair follicle stem cells. To investigate the contribution of hair follicle stem cells to skin wound healing in old *Med1^epi−/−^* mice, we performed a BrdU pulse-labeling experiment in 6-month-old *Med1^epi−/−^* mice and age-matched wild-type mice, 2 months before the wound creation. The analysis of BrdU label retaining cells in hair follicles adjacent to the wounds demonstrated that the number of BrdU-positive follicular slow-cycling cells was decreased in 6-month-old *Med1^epi−/−^* mice compared with age-matched wild-type mice (Figure 5C, p<0.01), indicating a significant depletion of hair follicle bulge stem cells in 6-month-old *Med1^epi−/−^* mice, which corresponds to our previous study [9]. Furthermore, BrdU-positive label retaining cells that had migrated into the epidermis adjacent to the wounds were detected in hair follicles of 6-month-old wild-type mice, while no such migrating cells were detected in 6-month-old *Med1^epi−/−^* mice (Figure 5C). This observation suggested that there is a distinct contribution of hair follicle bulge stem cells to the epidermal regenerative process in 6-month-old wild-type mice, but not in 6-month-old *Med1^epi−/−^* mice. On the other hand, the number of label retaining cells in hair follicles in 8-week-old *Med1^epi−/−^* mice was comparable with age-matched wild-type mice (Figure 5D).

## Discussion

In the present study, we investigated the effects of MED1 depletion in the epidermis on cutaneous wound healing in *Med1^epi−/−^* mice. Our results provide the first evidence that cutaneous wound healing is accelerated in 8-week-old *Med1^epi−/−^* mice compared with age-matched wild-type mice (Figure 1). The 8-week-old *Med1^epi−/−^* mice demonstrated a rapid re-epithelialization due to enhanced epidermal proliferation as well as migration in the wound sites but that was not due to the wound contraction (Figure 2). Although MED1 is known to function as a co-activator of nuclear receptors, such as PPAR, RXR and VDR, there has been no report demonstrating the accelerated wound healing phenotype in PPAR-KO mice, RXR-KO mice or VDR-KO mice [28]–[31]. Interestingly, *Med1^epi−/−^* keratinocytes show a significantly decreased expression of follistatin, a potent inhibitor of activin, with significantly increased MAPK activity compared with wild-type keratinocytes (Figure 3).

Activins, members of the TGF-β superfamily, are disulfide-linked dimeric proteins comprised of two β subunits. Three different forms of activin, homodimeric activin A (βAβA), homodimeric activin B (βBβB) and heterodimeric activin AB (βAβB), have been identified. Activins bind to heteromeric complexes of transmembrane receptor serine/threonine kinases, type I (ACVR1, 1B and 1C) and type II (ACVR2A and 2B) activin receptors [13], mediating their biological roles including the regulation of proliferation, differentiation, apoptosis, metabolism, homeostasis, immune function, endocrine function and wound repair in many tissues [32]. Follistatins, antagonists of activins, are soluble extracellular proteins consisting of varying molecular weight isoforms due to alternative splicing at the 3′ end of the mRNA [13], [33]–[35]. The most common isoforms of follistatin consist of 288 and 315 amino acids (FS288 and FS315, respectively). Follistatins have a higher affinity to the activin β subunits than the activin receptors [36]–[38] and inhibit the action of activins by two distinct mechanisms, as follows: 1) Membrane-bound follistatin FS288 has a high affinity to cell surface bound heparin sulfate, which causes the follistatin/activin complex to be internalized and subjected to lysosomal degradation. 2) The circulatory form of follistatin FS315, which contains a C-terminal acid tail, binds to activin and prevents binding to its receptors [13]. Although the precise distribution of the components of activin signaling in normal skin is uncertain, it is likely that the activinβA subunit is expressed in dermal cells while the activinβB subunit is expressed in proliferating keratinocytes at the wound edge and in the migrating epithelial tongue after injury [12]. On the other hand, follistatin mRNA is expressed mainly in the dermis and at low levels in the epidermis [12], [13].

Several studies using transgenic and knockout mice have clearly suggested the critical involvement of activins and follistatins during cutaneous wound healing. It has been reported that transgenic mice over-expressing the activinβA chain in keratinocytes showed an acceleration of the skin wound healing process with increased keratinocyte proliferation, hyperthickening of the tongue epithelium and excessive scar formation after skin injury [39], [40]. Additionally, it has been reported that mice without follistatin expression in keratinocytes (Fst null mice) show enhanced keratinocyte proliferation in the tail epidermis resulting in a thicker epithelium at the wound edge without excessive scarring after skin injury [41].

Previously it was reported that activins mediate wound repair after injury through the MAPK signaling pathway [15]–[17]. It has been reported that the blockade of JNK signaling by a JNK-specific inhibitor significantly suppresses keratinocyte proliferation at the wound site and subsequently delays wound closure [15], [16]. In our model, *Med1^epi−/−^* keratinocytes exhibit decreased follistatin expression and an increased activity of the MAPK pathway with or without the existence of exogenous activin A *in vitro*. Moreover, migration is enhanced in *Med1^epi−/−^* keratinocytes *in vitro* without exogenous activin A, while exogenous activin A elicits an increase in the percentage of *Med1^epi−/−^* keratinocytes in S-phase (Figure 3). After injury, 8-week-old *Med1^epi−/−^* mice show accelerated cutaneous wound healing without excessive granulation tissue formation (Figure 2). These findings suggest that in *Med1^epi−/−^* mice, the loss of follistatin expression in keratinocytes enhances the biological activity of activin secreted from keratinocytes and/or dermal fibroblasts and thus constitutively activates the MAPK signaling pathway in the epidermis, resulting in rapid wound healing just like in Fst null mice.

Several reports have suggested that MED1 and nuclear receptors are involved in regulating follistatin expression in various tissues. Necela et al. reported that activation of PPARγ down-regulates the expression of follistatin mRNA through dimerization with RXR in intestinal epithelial cells [42]. Matsumoto et al. reported that follistatin shows higher levels of expression in normal livers after partial hepatectomy but not in MED1-deficient livers, using transgenic mice [43]. Therefore, it is conceivable that MED1 depletion in keratinocytes has a direct and/or indirect effect on the expression of follistatin in our model, although the details are yet to be elucidated.

**Figure 6:**
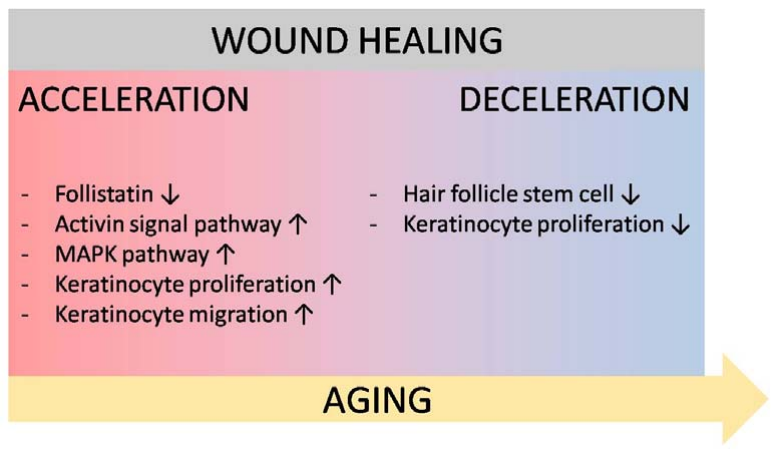
Proposed model of altered cutaneous wound healing in *Med1^epi−/−^* mice. In *Med1^epi−/−^* keratinocytes, the expression of follistatin is decreased. Consequently, activin, which is not sequestered by follistatin, is increased and activates the MAPK signaling pathway in keratinocytes in endocrine and/or paracrine manners. As a result, the proliferation and migration of *Med1^epi−/−^* keratinocytes are enhanced, contributing to rapid cutaneous wound healing process. However, as the number of hair follicle bulge stem cells decreases in old *Med1^epi−/−^* mice, the promoting effect of follistatin down-regulation on epidermal regeneration is overcome, resulting in impaired cutaneous wound healing.

Accumulating evidence indicates that there are multiple populations of epithelial stem cells locating in different parts of the epidermis [19], [20], [23], [24], [44]–[57]. They maintain normal skin homeostasis by regenerating the distinct epithelial cell lineages in the distinct parts of the epidermis as well as contribute to wound healing upon injury by recruiting undifferentiated progenitor cells to the wounded epidermis [21], [23]–[26]. Hair follicle stem cells do not normally contribute to epidermal homeostasis. However, after epidermal injury, hair follicle stem cells give rise to short-lived transient amplifying cells which are recruited into the wounded epidermis, facilitating the epidermal regeneration [27]. Hair follicles contain several populations of epithelial stem cells characterized by distinct expression patterns of stem cell markers, including CD34 and keratin 15 [19], [20]. Previously, we reported that the numbers of hair follicle stem cells which express CD34 and keratin 15 are reduced in *Med1^epi−/−^* mice from a few months to one year after birth, which suggests that MED1 plays a distinct role in the maintenance of hair follicle stem cells [9]. Correspondingly, skin wound healing in 6-month-old *Med1^epi−/−^* mice is significantly delayed with decreased numbers of Ki67-positive proliferating keratinocytes compared with age-matched wild-type mice (Figures 4, 5). The analysis of BrdU-positive label retaining cells further showed decreased numbers of hair follicle bulge stem cells migrating into the epidermis adjacent to the wound sites in 6-month-old *Med1^epi−/−^* mice, while no apparent change in 8-week-old *Med1^epi−/−^* mice, compared with age-matched wild-type mice (Figure 5). These findings indicate an impaired skin wound healing process due to the lack of CD34-positive and/or keratin 15-positive epithelial stem cells, which counteracts the positive effect of follistatin down-regulation on the wound healing in 6-month-old *Med1^epi−/−^* mice (shown schematically in Figure 6). To our knowledge, such a phenotype, in which cutaneous wound healing is accelerated in adolescence and is retarded in the elderly due to depletion of hair follicle stem cells, has not been previously reported and therefore is specific.

Although the precise mechanism by which MED1 depletion participates in the activin-follistatin system in keratinocytes and in hair follicle stem cell maintenance remains unclear, and the possibility that other factors involved in the wound healing process of *Med1^epi−/−^* skin can not be excluded, our findings shed light on a novel function of MED1 and offer possible new therapeutic approaches to target MED1 in the epidermis for cutaneous wound healing and aging.

## Materials and Methods

### Animals

The generation of *Med1^epi−/−^* mice, in which *Med1* is disrupted under control of the keratin 5 promoter, was described elsewhere [9]. All animal studies were performed according to protocols approved by the Institutional Animal Care and Use Committee at Osaka University. Mice that were used for wound healing study were housed appropriately as previously described [58]. Briefly, Mice were raised under light/dark (12-h/12-h) cycles and fed ad-libitum amount of standard chow and water according to the Institutional Animal Care and Use Committee at Osaka University. Mice were observed daily by the investigators and treated appropriately when displayed any signs of discomfort or illness by the facility veterinary.

### Wound Creation and Macroscopic Examination

Full-thickness wounds were made using a sterile biopsy punch with a diameter of 4 mm (NIPRO, Osaka, Japan) on the middle dorsal shaved telogen skin of *Med1^epi−/−^* mice and wild-type (*Med1^+/+^*) littermates at either 8 weeks or 6 months of age. Mice were administered sodium pentobarbital with or without sevoflurane anesthesia before wounding. The wounds were left uncovered and the animals were housed in separate cages. Wound healing was macroscopically monitored by digital photography at the indicated time points. The wound areas (percentage of wound area relative to the original wound) were calculated using the following formula: Relative open wound area (%) = [Open area on the indicated time point/Original wound area]×100.

### Immunohistochemistry

Immunohistochemical staining was performed as previously described [9]. In brief, 5 µm thick paraffin sections were deparaffinized and autoclaved in 10 mM sodium citrate (pH 6.0) for 15 min at 121°C to retrieve epitope structures. After washing in TBS-T (Tris-buffered saline with 1% Tween 20), the sections were treated with H_2_O_2_ and endogenous peroxidase activity was blocked. Specimens were then blocked with Protein Block Serum-Free (Dako, Glostrup, Denmark), incubated with rabbit polyclonal anti-Ki67 IgG (1∶500; Leica Microsystems, Buffalo Grove, IL), and mouse monoclonal anti-α-SMA IgG (1∶100; Dako) overnight at 4°C followed by incubation and visualization with a ChemMate ENVISION/HRP kit (Dako). Immunohistochemical staining for BrdU was performed using a BrdU In-Situ Detection kit (BD Bioscience, New Jersey, US) according to the manufacturer's protocol.

### Analysis of Re-Epithelialization and Wound Contraction

The width of each wound and the distance of the traversed epithelium were measured in H&E-stained sections at the indicated time points. The percentage of re-epithelialization was calculated according to the following formula: [distance of the minor axis covered by epithelium]/[distance of the minor axis between original wound edges]×100. The original wound edges were determined as the start sites of re-epithelialization (See Figure 1C). Wound contraction was estimated by measuring the distance of the minor axis between the original wound edges.

### Analysis of Cell Proliferation and Granulation Tissue

Ki67-positive cells were counted in the transitional epidermis and the epithelial tongue [2] of wounds and were related to the area of the same part of epidermis. The area of wound epidermis was determined using ImageJ software (National Institutes of Health, Bethesda, MD, USA). Myofibroblasts were identified by immunostaining of α-SMA in the granulation tissue and the stained area was determined by planimetric image analysis using ImageJ software.

### Isolation and Culture of Keratinocytes

Isolation and culture of keratinocytes was performed as previously described [9]. In brief, skins of newborn mice were derived after the mice had been sacrificed with excess anesthesia. Derived newborn mice skins were then treated with dispase and trypsin to separate the epidermis from the dermis. Isolated keratinocytes were then seeded on type I collagen coated dishes, and were cultured in CnT07 conditioned culture medium (KGM, CELLnTEC, Bern, Switzerland). For each experiment, keratinocytes were used as a primary culture or after one passage.

### Immunoblotting

Immunoblotting was performed as previously described [9]. Keratinocytes were cultured in keratinocyte basal medium (KBM, COSMO BIO, Tokyo, Japan) containing 0.03 mM calcium for 24 h and were washed and lysed in protein extraction buffer containing pH 7.2, 20 nmol/L HEPES with 1% Nonidet P-40, 0.4 M NaCl and aprotinin. Total protein extracts (10 µg/lane) were mixed with 2× SDS-PAGE buffer, and were heat denatured with 5% mercaptoethanol for 5 min at 80°C before loaded onto 4 to 12% gradient Tris-glycine gels (Invitrogen, Carlsbad, CA). After electrophoresis and transfer of proteins to nitrocellulose membranes, membranes were blocked in 5% milk in TBS-T buffer for 1 h, followed by 1 h incubation with rabbit polyclonal anti-follistatin IgG (sc-30194) (1∶200; Santa Cruz Biotechnology, Santa Cruz, CA), rabbit polyclonal anti-phospho-SAPK/JNK IgG (#9251) (1∶1000; Cell Signaling, Danvers, MA), rabbit polyclonal anti-SAPK/JNK IgG (#9252) (1∶1000; Cell Signaling), rabbit polyclonal anti-phospho-p44/42 MAPK (Erk1/2) (Thr202/Tyr204) IgG (#9101) (1∶1000; Cell Signaling), rabbit polyclonal anti-p44/42 MAPK (Erk1/2) IgG (#9102) (1∶1000; Cell Signaling) or anti-ββ-actin. After washing 3 times with TBS-T, membranes were incubated for 1 h with horseradish peroxidase conjugated secondary antibody. Visualization of the blots was performed using the ECL Plus Western Blotting Detection System (GE Healthcare, Buckinghamshire, UK). To investigate the phosphorylation of JNK and ERK by activin A treatment, after 24 h of supplement depletion, cultured keratinocytes were treated with or without 5 ng/mL recombinant human/mouse/rat activin A (R&D Systems, Minneapolis, MN) in KBM and were then harvested at 10, 30 and 60 min after the treatment. Quantification and densitometric analysis was performed using ImageJ software.

### Keratinocyte migration assay

For a wound healing assay, keratinocytes derived from skins of newborn *Med1^epi−/−^* mice and wild-type (*Med1^+/+^*) littermates were cultured in KBM and allowed to form confluent monolayers. After serum starvation for 24 h, keratinocytes were incubated with an S-phase cell cycle blocker mitomycin C (0.5 mg/ml) for 2 h. After straight scratch wounds were made with a p200 pipette tip, the suspended cells were removed washing with PBS and then incubated in KBM for 72 h. The number of cells which migrated into each wounded space was counted microscopically at the noted time intervals and related to the wounded area.

### Cell cycle analysis

For cell cycle analysis, keratinocytes derived from skins of newborn *Med1^epi−/−^* mice and wild-type (*Med1^+/+^*) littermates were seeded (2.5×10^5^) and cultured in KBM for 24 h to synchronize the cell cycles. Then, keratinocytes were treated with activin A (5 ng/mL) and BrdU (10 µM) and cultured in KBM for 24 h and harvested. The cell cycle of keratinocytes was analyzed by FACS CantoII(BD Biosciences) using a BD Pharmingen BrdU Flow kit (BD Biosciences) according to the manufacturer's protocol.

### BrdU labeling procedures

BrdU labeling of slow-cycling cells was performed as previously described [59]. Briefly, for BrdU labeling in 6-month-old mice, 4-month-old *Med1^epi−/−^* mice and wild-type (*Med1^+/+^*) littermates were intraperitoneally injected with BrdU (50 µg per g body weight) twice daily for 5 d and then conventional club hair plucking was performed. Eight weeks after plucking, wound creation and subsequent skin biopsy were performed and followed by immunohistochemistry for BrdU as described above. The number of BrdU-positive cells was counted in hair follicle sections with bulge region in 6-month-old *Med1^epi−/−^* mice and age-matched wild-type mice. For BrdU labeling in 8-week-old mice, neonatal mice were subcutaneously injected with BrdU (50 µg per g body weight) twice daily for 3 d from the third day after birth. After eight weeks, conventional club hair plucking and subsequent skin biopsy were performed and followed by immunohistochemistry for BrdU. The number of BrdU-positive cells was counted in hair follicle sections with bulge region in 8-week-old *Med1^epi−/−^* mice and age-matched wild-type mice.

### Statistical Analyses

An unpaired t-test was used to determine statistical significance when the values were normally distributed. An F-test was used to test if the variances are equal. When variances were significantly different according to the F-test, an unpaired t-test with Welch's correction was used.

## References

[1] Martin P (1997) Wound healing–aiming for perfect skin regeneration. Science 276: 75–81. 9082989 10.1126/science.276.5309.75

[2] Singer AJ, Clark RA (1999) Cutaneous wound healing. N Engl J Med 341: 738–746. 10471461 10.1056/NEJM199909023411006

[3] Gurtner GC, Werner S, Barrandon Y, Longaker MT (2008) Wound repair and regeneration. Nature 453: 314–321. 18480812 10.1038/nature07039

[4] Santoro MM, Gaudino G (2005) Cellular and molecular facets of keratinocyte reepithelization during wound healing. Exp Cell Res 304: 274–286. 15707592 10.1016/j.yexcr.2004.10.033

[5] Arwert EN, Hoste E, Watt FM (2012) Epithelial stem cells, wound healing and cancer. Nat Rev Cancer 12: 170–180. 22362215 10.1038/nrc3217

[6] Ge K, Guermah M, Yuan CX, Ito M, Wallberg AE, et al (2002) Transcription coactivator TRAP220 is required for PPAR gamma 2-stimulated adipogenesis. Nature 417: 563–567. 12037571 10.1038/417563a

[7] Zhu Y, Qi C, Jain S, Rao MS, Reddy JK (1997) Isolation and characterization of PBP, a protein that interacts with peroxisome proliferator-activated receptor. J Biol Chem 272: 25500–25506. 9325263 10.1074/jbc.272.41.25500

[8] Oda Y, Hu L, Bul V, Elalieh H, Reddy JK, et al (2012) Coactivator MED1 ablation in keratinocytes results in hair-cycling defects and epidermal alterations. J Invest Dermatol 132: 1075–1083. 22189783 10.1038/jid.2011.430 PMC3400544

[9] Nakajima T, Inui S, Fushimi T, Noguchi F, Kitagawa Y, et al (2012) Roles of MED1 in quiescence of hair follicle stem cells and maintenance of normal hair cycling. J Invest Dermatol in press. 10.1038/jid.2012.293 22931914

[10] Wankell M, Munz B, Hubner G, Hans W, Wolf E, et al (2001) Impaired wound healing in transgenic mice overexpressing the activin antagonist follistatin in the epidermis. Embo J 20: 5361–5372. 11574468 10.1093/emboj/20.19.5361 PMC125651

[11] Wankell M, Werner S, Alzheimer C, Werner S (2003) The roles of activin in cytoprotection and tissue repair. Ann N Y Acad Sci 995: 48–58. 12814938 10.1111/j.1749-6632.2003.tb03209.x

[12] Hubner G, Hu Q, Smola H, Werner S (1996) Strong induction of activin expression after injury suggests an important role of activin in wound repair. Dev Biol 173: 490–498. 8606007 10.1006/dbio.1996.0042

[13] McDowall M, Edwards NM, Jahoda CA, Hynd PI (2008) The role of activins and follistatins in skin and hair follicle development and function. Cytokine Growth Factor Rev 19: 415–426. 18922734 10.1016/j.cytogfr.2008.08.005

[14] Zhang L, Deng M, Parthasarathy R, Wang L, Mongan M, et al (2005) MEKK1 transduces activin signals in keratinocytes to induce actin stress fiber formation and migration. Mol Cell Biol 25: 60–65. 15601830 10.1128/MCB.25.1.60-65.2005 PMC538759

[15] Zhang M, Liu NY, Wang XE, Chen YH, Li QL, et al (2011) Activin B promotes epithelial wound healing in vivo through RhoA-JNK signaling pathway. PLoS One 6: e25143. 21949871 10.1371/journal.pone.0025143 PMC3176320

[16] Gazel A, Banno T, Walsh R, Blumenberg M (2006) Inhibition of JNK promotes differentiation of epidermal keratinocytes. J Biol Chem 281: 20530–20541. 16648634 10.1074/jbc.M602712200

[17] Zhang L, Wang W, Hayashi Y, Jester JV, Birk DE, et al (2003) A role for MEK kinase 1 in TGF-beta/activin-induced epithelium movement and embryonic eyelid closure. Embo J 22: 4443–4454. 12941696 10.1093/emboj/cdg440 PMC202382

[18] Fuchs E, Horsley V (2008) More than one way to skin. Genes Dev 22: 976–985. 18413712 10.1101/gad.1645908 PMC2732395

[19] Vidal VP, Chaboissier MC, Lutzkendorf S, Cotsarelis G, Mill P, et al (2005) Sox9 is essential for outer root sheath differentiation and the formation of the hair stem cell compartment. Curr Biol 15: 1340–1351. 16085486 10.1016/j.cub.2005.06.064

[20] Trempus CS, Morris RJ, Bortner CD, Cotsarelis G, Faircloth RS, et al (2003) Enrichment for living murine keratinocytes from the hair follicle bulge with the cell surface marker CD34. J Invest Dermatol 120: 501–511. 12648211 10.1046/j.1523-1747.2003.12088.x

[21] Levy V, Lindon C, Zheng Y, Harfe BD, Morgan BA (2007) Epidermal stem cells arise from the hair follicle after wounding. Faseb J 21: 1358–1366. 17255473 10.1096/fj.06-6926com

[22] Nowak JA, Polak L, Pasolli HA, Fuchs E (2008) Hair follicle stem cells are specified and function in early skin morphogenesis. Cell Stem Cell 3: 33–43. 18593557 10.1016/j.stem.2008.05.009 PMC2877596

[23] Snippert HJ, Haegebarth A, Kasper M, Jaks V, van Es JH, et al (2010) Lgr6 marks stem cells in the hair follicle that generate all cell lineages of the skin. Science 327: 1385–1389. 20223988 10.1126/science.1184733

[24] Mardaryev AN, Meier N, Poterlowicz K, Sharov AA, Sharova TY, et al (2011) Lhx2 differentially regulates Sox9, Tcf4 and Lgr5 in hair follicle stem cells to promote epidermal regeneration after injury. Development 138: 4843–4852. 22028024 10.1242/dev.070284 PMC4067271

[25] Blanpain C (2010) Stem cells: Skin regeneration and repair. Nature 464: 686–687. 20360726 10.1038/464686a

[26] Lau K, Paus R, Tiede S, Day P, Bayat A (2009) Exploring the role of stem cells in cutaneous wound healing. Exp Dermatol 18: 921–933. 19719838 10.1111/j.1600-0625.2009.00942.x

[27] Ito M, Liu Y, Yang Z, Nguyen J, Liang F, et al (2005) Stem cells in the hair follicle bulge contribute to wound repair but not to homeostasis of the epidermis. Nat Med 11: 1351–1354. 16288281 10.1038/nm1328

[28] Bikle DD, Elalieh H, Chang S, Xie Z, Sundberg JP (2006) Development and progression of alopecia in the vitamin D receptor null mouse. J Cell Physiol 207: 340–353. 16419036 10.1002/jcp.20578

[29] Li M, Chiba H, Warot X, Messaddeq N, Gerard C, et al (2001) RXR-alpha ablation in skin keratinocytes results in alopecia and epidermal alterations. Development 128: 675–688. 11171393 10.1242/dev.128.5.675

[30] Cianferotti L, Cox M, Skorija K, Demay MB (2007) Vitamin D receptor is essential for normal keratinocyte stem cell function. Proc Natl Acad Sci U S A 104: 9428–9433. 17517646 10.1073/pnas.0702884104 PMC1890511

[31] Peters JM, Lee SS, Li W, Ward JM, Gavrilova O, et al (2000) Growth, adipose, brain, and skin alterations resulting from targeted disruption of the mouse peroxisome proliferator-activated receptor beta(delta). Mol Cell Biol 20: 5119–5128. 10866668 10.1128/mcb.20.14.5119-5128.2000 PMC85961

[32] Chen YG, Wang Q, Lin SL, Chang CD, Chuang J, et al (2006) Activin signaling and its role in regulation of cell proliferation, apoptosis, and carcinogenesis. Exp Biol Med (Maywood) 231: 534–544. 16636301 10.1177/153537020623100507

[33] Shimasaki S, Koga M, Esch F, Cooksey K, Mercado M, et al (1988) Primary structure of the human follistatin precursor and its genomic organization. Proc Natl Acad Sci U S A 85: 4218–4222. 3380788 10.1073/pnas.85.12.4218 PMC280398

[34] Shimasaki S, Koga M, Esch F, Mercado M, Cooksey K, et al (1988) Porcine follistatin gene structure supports two forms of mature follistatin produced by alternative splicing. Biochem Biophys Res Commun 152: 717–723. 3365249 10.1016/s0006-291x(88)80097-4

[35] Inouye S, Guo Y, DePaolo L, Shimonaka M, Ling N, et al (1991) Recombinant expression of human follistatin with 315 and 288 amino acids: chemical and biological comparison with native porcine follistatin. Endocrinology 129: 815–822. 1906804 10.1210/endo-129-2-815

[36] Shimonaka M, Inouye S, Shimasaki S, Ling N (1991) Follistatin binds to both activin and inhibin through the common subunit. Endocrinology 128: 3313–3315. 2036994 10.1210/endo-128-6-3313

[37] de Winter JP, ten Dijke P, de Vries CJ, van Achterberg TA, Sugino H, et al (1996) Follistatins neutralize activin bioactivity by inhibition of activin binding to its type II receptors. Mol Cell Endocrinol 116: 105–114. 8822271 10.1016/0303-7207(95)03705-5

[38] Wang Q, Keutmann HT, Schneyer AL, Sluss PM (2000) Analysis of human follistatin structure: identification of two discontinuous N-terminal sequences coding for activin A binding and structural consequences of activin binding to native proteins. Endocrinology 141: 3183–3193. 10965889 10.1210/endo.141.9.7675

[39] Munz B, Smola H, Engelhardt F, Bleuel K, Brauchle M, et al (1999) Overexpression of activin A in the skin of transgenic mice reveals new activities of activin in epidermal morphogenesis, dermal fibrosis and wound repair. Embo J 18: 5205–5215. 10508154 10.1093/emboj/18.19.5205 PMC1171591

[40] Munz B, Tretter YP, Hertel M, Engelhardt F, Alzheimer C, et al (2001) The roles of activins in repair processes of the skin and the brain. Mol Cell Endocrinol 180: 169–177. 11451588 10.1016/s0303-7207(01)00514-7

[41] Antsiferova M, Klatte JE, Bodo E, Paus R, Jorcano JL, et al (2009) Keratinocyte-derived follistatin regulates epidermal homeostasis and wound repair. Lab Invest 89: 131–141. 19079322 10.1038/labinvest.2008.120 PMC4087116

[42] Necela BM, Su W, Thompson EA (2008) Peroxisome proliferator-activated receptor gamma down-regulates follistatin in intestinal epithelial cells through SP1. J Biol Chem 283: 29784–29794. 18768463 10.1074/jbc.M804481200 PMC2662057

[43] Matsumoto K, Yu S, Jia Y, Ahmed MR, Viswakarma N, et al (2007) Critical role for transcription coactivator peroxisome proliferator-activated receptor (PPAR)-binding protein/TRAP220 in liver regeneration and PPARalpha ligand-induced liver tumor development. J Biol Chem 282: 17053–17060. 17438330 10.1074/jbc.M701956200

[44] de Lau W, Barker N, Low TY, Koo BK, Li VS, et al (2011) Lgr5 homologues associate with Wnt receptors and mediate R-spondin signalling. Nature 476: 293–297. 21727895 10.1038/nature10337

[45] Brownell I, Guevara E, Bai CB, Loomis CA, Joyner AL (2011) Nerve-derived sonic hedgehog defines a niche for hair follicle stem cells capable of becoming epidermal stem cells. Cell Stem Cell 8: 552–565. 21549329 10.1016/j.stem.2011.02.021 PMC3089905

[46] Garza LA, Yang CC, Zhao T, Blatt HB, Lee M, et al (2011) Bald scalp in men with androgenetic alopecia retains hair follicle stem cells but lacks CD200-rich and CD34-positive hair follicle progenitor cells. J Clin Invest 121: 613–622. 21206086 10.1172/JCI44478 PMC3026732

[47] Blanpain C, Lowry WE, Geoghegan A, Polak L, Fuchs E (2004) Self-renewal, multipotency, and the existence of two cell populations within an epithelial stem cell niche. Cell 118: 635–648. 15339667 10.1016/j.cell.2004.08.012

[48] Jensen UB, Yan X, Triel C, Woo SH, Christensen R, et al (2008) A distinct population of clonogenic and multipotent murine follicular keratinocytes residing in the upper isthmus. J Cell Sci 121: 609–617. 18252795 10.1242/jcs.025502 PMC2963074

[49] Morris RJ, Liu Y, Marles L, Yang Z, Trempus C, et al (2004) Capturing and profiling adult hair follicle stem cells. Nat Biotechnol 22: 411–417. 15024388 10.1038/nbt950

[50] Tumbar T, Guasch G, Greco V, Blanpain C, Lowry WE, et al (2004) Defining the epithelial stem cell niche in skin. Science 303: 359–363. 14671312 10.1126/science.1092436 PMC2405920

[51] Rhee H, Polak L, Fuchs E (2006) Lhx2 maintains stem cell character in hair follicles. Science 312: 1946–1949. 16809539 10.1126/science.1128004 PMC2405918

[52] Frye M, Fisher AG, Watt FM (2007) Epidermal stem cells are defined by global histone modifications that are altered by Myc-induced differentiation. PLoS One 2: e763. 17712411 10.1371/journal.pone.0000763 PMC1945016

[53] Jensen KB, Collins CA, Nascimento E, Tan DW, Frye M, et al (2009) Lrig1 expression defines a distinct multipotent stem cell population in mammalian epidermis. Cell Stem Cell 4: 427–439. 19427292 10.1016/j.stem.2009.04.014 PMC2698066

[54] Horsley V, Aliprantis AO, Polak L, Glimcher LH, Fuchs E (2008) NFATc1 balances quiescence and proliferation of skin stem cells. Cell 132: 299–310. 18243104 10.1016/j.cell.2007.11.047 PMC2546702

[55] Jaks V, Barker N, Kasper M, van Es JH, Snippert HJ, et al (2008) Lgr5 marks cycling, yet long-lived, hair follicle stem cells. Nat Genet 40: 1291–1299. 18849992 10.1038/ng.239

[56] Nguyen H, Merrill BJ, Polak L, Nikolova M, Rendl M, et al (2009) Tcf3 and Tcf4 are essential for long-term homeostasis of skin epithelia. Nat Genet 41: 1068–1075. 19718027 10.1038/ng.431 PMC2792754

[57] Uchugonova A, Duong J, Zhang N, Konig K, Hoffman RM (2011) The bulge area is the origin of nestin-expressing pluripotent stem cells of the hair follicle. J Cell Biochem 112: 2046–2050. 21465525 10.1002/jcb.23122

[58] Castilho RM, Squarize CH, Leelahavanichkul K, Zheng Y, Bugge T, et al (2010) Rac1 is required for epithelial stem cell function during dermal and oral mucosal wound healing but not for tissue homeostasis in mice. PLoS One 5: e10503. 20463891 10.1371/journal.pone.0010503 PMC2865533

[59] Ito M, Kizawa K, Toyoda M, Morohashi M (2002) Label-retaining cells in the bulge region are directed to cell death after plucking, followed by healing from the surviving hair germ. J Invest Dermatol 119: 1310–1316. 12485433 10.1046/j.1523-1747.2002.19644.x

